# Cross-sectional Study on the Effects of Socioeconomic Factors on Lead Exposure in Children by Gender in Serpong, Indonesia 

**DOI:** 10.3390/ijerph9114135

**Published:** 2012-11-14

**Authors:** Dewi U. Iriani, Takehisa Matsukawa, Muhammad K. Tadjudin, Hiroaki Itoh, Kazuhito Yokoyama

**Affiliations:** 1 Department of Epidemiology and Environmental Health, Juntendo University Faculty of Medicine, 2-1-1 Hongo, Bunkyo-ku, Tokyo 113-8421, Japan; Email: dewi@juntendo.ac.jp (D.I.); tmatsuka@juntendo.ac.jp (T.M.); h-itou@juntendo.ac.jp (H.I.); kyokoya@juntendo.ac.jp (K.Y.); 2 Faculty of Medicine and Health Sciences, Syarif Hidayatullah, State Islamic University, Jl, Kertamukti, Ciputat, Jakarta 15412, Indonesia; Email: tajudin@dnet.net.id

**Keywords:** lead, children, socioeconomic factors, drinking water, sex differences, Indonesia

## Abstract

To elucidate the socioeconomic factors influencing lead exposure in elementary school children by gender, 108 children (56 male, 52 female), aged 6–7 years, were randomly selected from 39 elementary state schools in Serpong, Banten, Indonesia. Their parents were interviewed to obtain information on sociodemographic characteristics. Their blood lead (BPb) levels were measured by atomic absorption spectrophotometry. BPb concentrations were significantly higher in males than in females, *i.e.*, 6.8 ± 2.0 (2.9–12.5) µg/dL and 5.9 ± 1.9 (3.1–11.7) µg/dL, respectively (*p* < 0.05). Lower socioeconomic status and well water use were associated with increased BPb concentrations, especially in females. The proportion of well water use was related to lower socioeconomic status. Lower socioeconomic status linked with well water drinking seemed to be associated with increased lead exposure in children in Serpong. Their exposure levels possibly varied according to gender differences in behavior. An intervention should be instituted among children in Serpong with BPb concentrations of 10 µg/dL or above.

## 1. Introduction

Recently, there has been growing interest in the health effects of long-term exposure to low levels of lead, which can only be observed at a subclinical level and thus are not evident in routine medical examinations. Children are believed to be more sensitive to lead toxicity than adults, as it is reported that children absorb 40–50% of dietary lead and retain 2–25%, whereas adults absorb 5–10% and retain little [1]. The most serious health consequence of lead exposure is its effects on the central nervous system [2,3,4]. Many large-scale studies clearly demonstrated that low levels of lead exposure were associated with a decrease in the intelligence quotient (IQ) in children [5,6,7,8,9]. This is thought to result from lead affecting the development of the central nervous system, causing neurological impairment even at low concentrations [10]. For this reason, measures against lead poisoning, such as banning of leaded gasoline, have been implemented in developed countries since the 1980s, and consequently the blood lead (BPb) levels of children have declined along with a decrease in atmospheric concentrations of lead [11]. On 4 January 2012, the Advisory Committee on Childhood Lead Poisoning Prevention recommended a “reference value” of 5 µg/dL which was the 97.5th percentile of the BPb concentration distribution in US children aged 1–5 years, eliminating the use of a BPb concentration of 10 µg/dL as the “blood lead level of concern” [12]. 

To take appropriate action against lead exposure among children, it is important to elucidate significant determinants of the exposure. Many studies have demonstrated that socioeconomic status (SES) influences BPb concentrations in children. For example, children living below the poverty line and having parents with lower education are associated with higher BPb concentrations [13,14,15]. For the population without occupational exposure, drinking water is one of the important sources of exposure to lead. The United States Environmental Protection Agency has estimated that 14–20% of total childhood lead exposure in the United States is from drinking water [16]. Some studies have shown higher BPb concentrations in male than in female children [17,18,19,20], which is explained by differences in playing and mouthing behavior [21] and playtime activities [19]. However, gender differences in the effects of SES on lead exposure remain to be investigated. 

In rapidly developing Asian countries, the suburbs of big cities have serious environmental pollution problems, not only because of expanding urban areas and thus increases in traffic, but also because of the lack of sufficient monitoring or socioeconomic analysis of environmental pollution. In Indonesia, the Jakarta metropolitan area has faced serious air pollution problems, caused mainly by emissions from the rapidly increasing number of cars. The Indonesian Government banned the use of leaded gasoline in Jakarta in 2001, and then in all cities of Indonesia in 2006 [22]. According to surveys conducted later, lead in the ambient air and BPb concentrations in elementary school students were reduced in Jakarta [23]. 

Syafrul *et al.* [24] reported that the concentrations of atmospheric lead were higher in Serpong district, a suburban area located 25 km southwest of Jakarta, than in Jakarta itself, and that BPb concentrations in Serpong children were 7–12 µg/dL in 2005. However, no subsequent studies have been conducted to assess lead exposure in children after the ban of leaded gasoline. Therefore, the aim of the present study was to measure BPb concentrations in children and to identify whether SES affects their lead exposure, with attention to gender differences. 

## 2. Methods

### 2.1. Subjects

A cross-sectional study was conducted in children aged 6–7 years in Serpong, Banten, Indonesia, during the period from April to May 2011. Children of these ages were targeted because previous studies [7,8,9] reported significant adverse effects of low level lead exposure on cognitive function in children aged 6–7 years old.

From 39 elementary state schools in Serpong, 200 children aged 6–7 years were randomly selected. Parents were approached and informed about the study 1 month before it started. Of these, 116 parents gave informed consent for blood sampling from their children and consented to be interviewed. During the study period, blood samples of 110 children were collected and two children were excluded because they were older than 7 years. Thus, 108 children were finally included in the study. 

The study protocol was reviewed and approved by the ethical committee of Juntendo University, Japan (22041), and the National Institute of Health Research and Development, Indonesian Ministry of Health (LB.03.02/KE/6937/2020).

### 2.2. Collection and Analysis of Blood Samples

BPb concentrations were measured as in a previous study of our laboratory [25]. A 5 mL blood sample from each child was collected from the radial vein in the morning by local public health staff, and stored in demineralized and heparinized tubes in a refrigerator set below 10°C. For analysis, blood samples were equilibrated to room temperature and 100 µL aliquots were diluted by solvent containing 1% Triton-X 100 and 0.5% nitric acid. BPb concentrations were determined using a flameless atomic absorption spectrophotometer (Hitachi Z-2000, Tokyo, Japan). Standard solutions for calibration were 0, 5, 10, and 15 µg/dL, prepared by mixing standard lead solution and 0.5% nitric acid. The matrix modifier was made up of 0.5% ammonium dihydrophosphate and 0.5% Triton-X 100. Every measurement was conducted in duplicate. The detection limit for lead was 0.01 µg/dL.

### 2.3. Questionnaire

A questionnaire on the socioeconomic characteristics of the family, medical and nutritional history, and potential environmental and household sources of exposure to lead (e.g., distance from house to industrial plant, traffic, parental occupation, water drinking sources, plastic toy use, traditional medicine and canned food/drink consumption, and paint in the home) was prepared for interview of the parents by trained interviewers from Syarif Hidayatullah State Islamic University, Jakarta. The subjects’ parents had face-to-face interviews at their homes or at their child’s school. The children’s body weights were measured using a bathroom scale to an accuracy of 1 kg, and heights were measured using a standard mechanical stadiometer to an accuracy of 1 cm. Body mass index (BMI, kg/m^2^) was calculated using body weight and height.

### 2.4. Statistical Analysis

Statistical analysis was performed using SPSS 19.00. A common logarithm of BPb (log BPb) was used in all statistical analyses because the distribution of BPb was skewed. Analyses were performed separately for male and female children. Pearson correlation coefficients of BPb concentrations with numeric variables of sociodemographic characteristics of children and parents were calculated. The Student *t*-test was used to examine the differences in BPb concentrations according to sociodemographic characteristics of children and parents, and home environment factors. 

The Chi-square test was conducted to examine the relationship between socioeconomic characteristics and well water use. In this analysis, the variables which had more than two categories were dichotomized. The categories of father’s or mother’s education were grouped into “low” (elementary school and junior high school) and “high” (senior high school and diploma/university). Father’s income was grouped into “low” (3,000,000 Rupiahs or less) and “high” (>3,000,000 Rupiahs), and father’s job was grouped into “unskilled” (elementary occupation, service and sales workers, craft and related trade workers, plant machine operators and assemblers) and “skilled” (managers, professionals, technician and associated professionals, clerical support workers, armed forces occupations).

To further confirm univariate analyses observations, we performed multivariate linear regression analysis to assess predictors of BPb concentration as socioeconomic status might be confounding factors on lead exposure [26]. Multivariate logistic regression analyses were also performed to examine the factors associated with increased odds of having BPb concentration above the median (6.42, 5.46, and 6.05 µg/dL for males, females, and both combined, respectively). In this analysis, socioeconomic characteristics variables which were significantly related to BPb concentrations by the Student t-test were used as independent variables. 

## 3. Results

**Figure 1:**
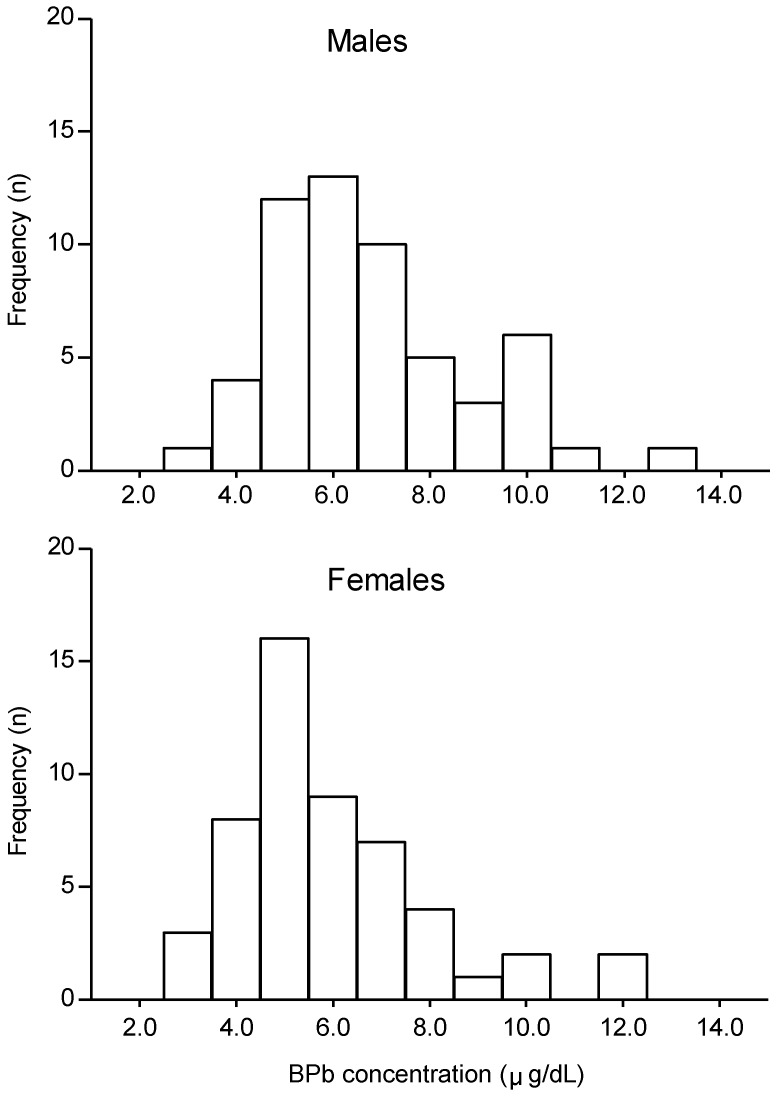
Distribution of blood lead (BPb) concentrations in 6–7-year-old children (n = 108, 56 male, 52 female) in Serpong, Banten, Indonesia.

Among the 108 children examined, 56 were male and 52 were female. BPb concentrations are shown in Figure 1 for males and females separately. The mean ± standard deviation (range) of BPb concentrations were 6.4 ± 2.0 (2.9–12.5) µg/dL, for all children, 6.8 ± 2.0 (2.9–12.5)µg/dL for males and 5.9 ± 1.9 (3.1–11.7) µg/dL for females. BPb concentrations were significantly higher in males than in females (*p* < 0.05). Eight children (7.5%) had BPb of ≥ 10 µg/dL, with the highest value being 12.5 µg/dL.

Approximately 2% of mothers and 60% of fathers smoked in the house. A large proportion of fathers (61.6%) were educated to senior high school level or above, whereas this proportion was lower in mothers (42.6%). The monthly income was 1,000,000 Rupiahs (approximately 100 US$) or above in approximately 80% of fathers, while most mothers (77.8%) earned less than 1,000,000 Rupiahs per month. The largest proportion of fathers (27.8%) were service and sales workers, and the majority of mothers were unemployed (58.3%).The time to school and distance between house and school were 10.1 ± 7.2 (2–60) min and 876.1 ± 903.3 (3–6,000) m, respectively. The majority of children went to school on foot (44.4%), drank well water (50.0%), and lived in homes which had been painted (91.7%). The characteristics of children, socioeconomic characteristics of parents, and the home environmental conditions of children are shown in Appendix Table A1, Table A2 and Table A3, respectively.

**Figure 2:**
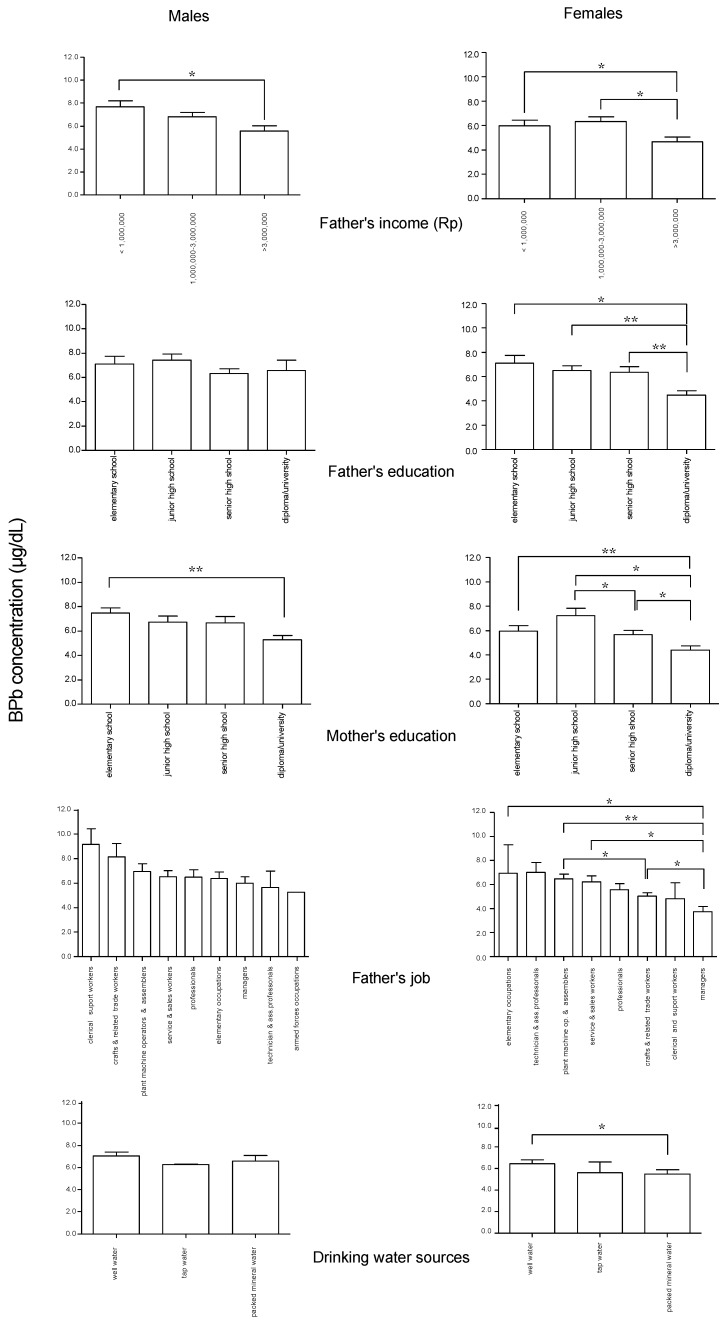
Significant differences in BPb concentrations according to socioeconomic characteristics in the male and female children. Rp: Rupiahs. Means with standard error bars. * *p* < 0.05, ** *p* < 0.01.

Significant differences in BPb concentrations in children according to socioeconomic characteristics of their parents and their home environmental conditions are shown in Figure 2. In both male and female children, father’s income < 1,000,000 Rupiahs was associated with higher BPb concentrations. Similarly, BPb concentrations were higher in children whose mothers were educated only to elementary school level, compared with mothers with a diploma or university education. In female children, BPb concentrations were higher if their father’s education level was only to elementary school level, and when the father was a craft and related trade worker, service and sales worker, plant machine operator and assembler, or elementary worker compared with a father who was a manager. When the father was a plant machine operator and assembler, BPb concentrations in female children were higher than when the father was a craft and related trade worker. Female children who drank well water showed higher BPb concentrations than those who drank bottled mineral water.

Table 1 shows the differences in well water use by socioeconomic characteristics. In both male and female children, the proportion of well water use was higher when the father’s job was unskilled. This proportion was higher when the father’s income was low in females, and when the mother’s education level was low in males.

As shown in Table 2, only gender was significantly related to BPb concentration in the multiple linear regression. The results of multivariate logistic analysis (Table 3) showed that male children were approximately three times more likely to have higher BPb concentration than female children. Although not statistically significant, father’s income, father’s education, mother’s education, and father’s job were related to increased BPb concentration in both male and female children. Children whose father had skilled job were less likely to have higher BPb concentration than children whose father had unskilled job.

**Table 1:** Differences in well water use by socioeconomic characteristics in the study children.

Parent’s characteristics *^a^*	Males	Females
Father’s income		
Low	25 (86)	25 (100) *
High	4 (14)	0 (0)
Father’s education		
Low	14 (48)	14 (56)
High	15 (52)	11 (44)
Mother’s education		
Low	22 (76) *	17 (68)
High	7 (24)	8 (32)
Father’s job		
Unskilled	25 (86) *	23 (92) *
Skilled	4 (14)	2 (8)

^a^ number (%), * *p* < 0.05 (Chi-square test).

**Table 2:** Predictors of log BPb among children in Serpong: Multivariate linear regression analysis.

	Standardized regression coefficient		
	Male (n = 56)	Female (n = 52)	Total (n = 108)
Independent variables			
Gender			0.200 *
Father’s income (Rupiahs)	−0.013	0.122	0.157
Father’s education	0.117	−0.010	0.051
Mother’s education	0.204	0.028	0.186
Father’s job	0.009	0.200	−0.054
Drinking water sources	0.033	0.167	0.107
Adjusted R square	0.173	0.064	0.177 *

* *p* < 0.05. Gender: 0 = female, 1 = male; Father’s income: 1 = >3,000,000, 2 = 1,000,000–3,000,000, 3 = <1,000,000. Father’s and mother’s education: 1 = diploma/university, 2 = senior high school, 3 = junior high school, 4 = elementary school; Father’s job: 0 = skilled, 1 = unskilled; Drinking water sources:0 = non-well, 1 = well.

**Table 3:** Factors associated with increased BPb concentration among children in Serpong: Multivariate logistic regression analysis.

Independent Variables		Odds Ratio [95 % Confidence Interval]		
		Male (n = 56)	Female (n = 52)	Total (n = 108)
Gender				
	Female			1
	Male			2.627 [1.139–6.056] *
Father’s income (Rupiahs)				
	>3,000,000	1	1	1
	1,000,000–3,000,000	1.991 [0.320–12.389]	2.958 [0.196–44.736]	2.557 [0.643–10.165]
	< 1,000,000	6.466 [0.592–70.629]	1.552 [0.071–34.033]	3.072 [0.588–16.057]
Father’s education				
	High education	1	1	1
	Low education	1.686 [0.385–7.383]	1.102 [0.257–4.715]	1.425 [0.511–3.970]
Mother’s education				
	High education	1	1	1
	Low education	1.730 [0.340–8.812]	2.435 [0.514–11.538]	1.926 [0.661–5.610]
Father’s job				
	Skilled	1	1	1
	Unskilled	0.153 [0.024–0.981]	0.522 [0.081–3.366]	0.262 [0.075–0.915] *
Drinking water sources				
	Non-well water	1	1	1
	Well	1.806 [0.474–6.887]	3.491 [0.815–14.953]	2.515 [0.984–6.427]

* *p* < 0.05, 1 = reference. Below (0) or above (1) median (6.42, 5.46, and 6.05 µg/dL for males, females, and both combined respectively). Father’s and mother’s education: 0 = high education (diploma/university and senior highs school) 1 = low education (junior high school and elementary school); Father’s job: 0 = skilled (managers, professionals, technician and associated professionals, clerical support workers, armed forces occupations), 1 = unskilled (elementary occupation, service and sales workers, craft and related trade workers, plant machine operators and assemblers).

## 4. Discussion

BPb concentrations of children in Serpong in 2011 were not greatly different from those measured in 2005 [24], indicating that lead exposure among children in Serpong had not decreased despite the ban of leaded gasoline in Indonesia in 2006. Soil dust, drinking water, and food products are the possible sources of lead exposure in this population [27]. The fact that half of the subjects obtained drinking water from wells likely contributes to lead exposure, as it was observed that higher BPb concentrations were associated with well water use in female children. In our unpublished data, lead concentrations in two water samples collected from three wells in Serpong (0.001, 0.02 and 0.05 mg/L) exceeded the World Health Organization limit (0.01 mg/L in drinking water) [28]. In support of this, lead concentrations of the Cisadane River that runs through the Serpong area are reported to be in the range from 20 to 30 ppm [29,30], and the ground soil in the area along the river was reported to contain high levels of lead [29]. The present study also indicated that well water use was associated with lower SES. Monitoring of lead in the local environment and public health measures on drinking water management are needed to reduce and to prevent lead exposure in the future. 

The present study also revealed the gender difference in BPb concentrations in children, with higher values in males. This was also demonstrated by the multivariate analyses (Table 2 and Table 3). This finding is compatible with the studies conducted in Krakow, Thailand, China, and Bangladesh [17,18,19,20]. However, the differences between BPb levels in male and female children were typically less than 10% [21]. The gender difference in BPb levels in the present study might have been caused by differences in play behavior between male and female children. Compared with female children, male children tend to spend more time outdoors, likely ingesting soil dust contaminated with lead through hands and mouth [18,19,21]. 

In male and female children, BPb concentrations were significantly affected by father’s income and mother’s education. In female children, BPb concentrations also showed significant differences according to father’s education and father’s job. Thus, BPb concentrations in female children seem to have been affected by SES more than in male children. As higher BPb concentrations were observed in male children, factors other than those examined in the present study are likely to have contributed to their lead exposure levels. Although previous studies [13,14,15,18,31] on the relationships between lead exposure and SES did not refer to gender differences, taking gender differences into account could be important in preventive measures against lead toxicity in children. 

The multivariate analyses failed to demonstrate statistically significant results for the above findings in the present study (Table 2 and Table 3). This could be resulted from a relatively small number of subjects; a larger scale epidemiological study should be necessary. In the multivariate logistic analyses, in addition, there was a statistically significant association between father in skilled job and incereased BPb concentration. This tendency did not match those of other studies [15,18,19,31]. In the present study, father’s job were categorized based on the International Standard Classification of Occupations 1988. The definition, however, in terms of father’s job should be considered further. As many studies demonstrated that low level lead exposure adversely affects cognitive function among children [5,6,7,8,9], it is necessary in a future study to recruit a larger number of subjects from these schools to know the effects of lead on their cognitive function. Furthermore, other potential sources of lead, such as in house dust, soil, air, toys, and food should also be examined.

## 5. Conclusions

Lower socioeconomic status associated with well water-drinking increased the exposure of children in Serpong to lead. Gender differences in exposure levels were possibly related to differences in behavior of the two genders. Additional testing and intervention is warranted among children in Serpong with BPb concentrations of 10 µg/dL or above.

## Appendix

**Table A1:** Characteristics of the study children.

Variable ^a^		Males	Females	Total
Age (year)		7.1 (0.5, 6.1–7.9)	6.9 (0.5, 6.0–7.8)	7.0 (0.5, 6.0–7.9)
Weight (kg)		21.9 (4.7, 15.0–37.0)	20.6 (4.8, 11.5–39.0)	21.3 (4.8, 11.5–39.0)
Height (cm)		118.6 (5.8, 107–137)	116.3 (5.2, 105–132)	117.5 (5.6, 105–137)
Birth weight (g)		3,060.70	3,158.10	3,107.60
		(559.7, 1,500–4,300)	(514.9, 1,400–4,500)	(538.3, 1,400–4,500)
BMI		21.9 (4.7, 15.0–37.0)	20.6 (4.8, 11.5–39.0)	21.3 (4.7, 11.5–39.0)
Birth order		1.9 (1.2, 1–5)	1.9 (1.2, 1–7)	1.9 (1.2, 1–7)
Number of children in family		2.4 (1.3, 1–6)	2.4 (1.2, 1–7)	2.4 (1.2, 1–7)
Milk consumption		31 (55.4 %)	29 (55.8 %)	60 (55.6 %)
Volume (mL)		200.4 (52.5, 0–1,000)	222.5 (267.9, 0–900)	211.02 (259.07, 0–1,000)
Breakfast				
	No	7 (12.5%)	6 (11.5%)	13 (12.0%)
	Sometimes	13 (23.2 %)	18 (34.6 %)	31 (28.7 %)
	Yes	36 (64.3 %)	28 (53.8 %)	64 (59.3 %)
Breastfeeding history				
	All	30 (53.6%)	27 (51.9%)	57 (52.8%)
	Mostly	10 (17.9%)	11 (21.2%)	21 (19.4%)
	Partially	9 (16.1%)	10 (19.2%)	19 (17.6%)
	None	7 (12.5%)	4 (7.7%)	11 (10.2%)

^a ^mean (standard deviation (SD), range) or number (%).

**Table A2:** Socioeconomic characteristics of parents of the study children.

Variable ^a^	Males	Females	Total
Age (years)			
Father	39.1 (7.8, 27–65)	38.6 (7.1, 28–67)	33.6 (5.8, 24–47)
Mother	33.0 (7.0, 22–54)	33.6 (5.8, 24–47)	33.3 (6.4, 22–54)
Weight (kg)			
Father	65.8 (8.6, 45.0–90.0)	62.6 ( 8.5,45.0–85.0)	64.3 (8.7, 45.0–90.0)
Mother	55.8 (10.7, 35.0–90.0)	55.4 (8.1, 37.0–78.0)	55.6 (9.5, 35.0–90.0)
Height (cm)			
Father	165.9 (6.5, 135.0–170.0)	166.7 (6.6, 150.0–183.0)	166.3 (6.6, 135.0–183.0)
Mother	155.3 (5.7, 141.0–170.0)	154.8 (6.3, 140.0–165.0)	155.1 (5.9, 140.0–170.0)
Father’s income (Rupiahs)			
<1,000,000	12 (21.4%)	11 (21.2%)	23 (21.3%)
1,000,000–3,000,000	35 (62.5%)	31 (59.6%)	66 (61.1%)
>3,000,000	9 (16.1%)	10 (19.2%)	19 (17.6%)
Education			
Father			
Elementary school	11 (19.6%)	8 (15.4%)	19 (17.6%)
Junior high school	14 (25%)	9 (17.31%)	23(21.3%)
Senior high school	22 (39.3%)	24 (46.2%)	46 (42.6%)
Diploma/University	9 (16.1%)	11 (21.2%)	20 (18.5%)
Mother			
Elementary school	15 (26.5%)	15 (28.8%)	30 (27.8%)
Junior high school	18 (32.1%)	14 (26.9%)	32 (29.6%)
Senior high school	18 (32.1%)	14 (26.9%)	32 (29.6%)
Diploma/University	5 (8.9%)	9 (17.3%)	14 (13.0%)
Smoking status			
Father			
Smoke at home	34 (60.7%)	32 (61.5%)	66 (61.1%)
Smoke but not at home	4 (7.1%)	8 (15.4%)	12 (11.1%)
Ex-smoker	6 (10.7%)	1 (1.9%)	7 (6.5%)
Never	12 (21.4%)	11 (21.2%)	23 (21.3%)
Mother			
Smoke at home	1 (1.8%)	1 (1.9%)	2 (1.9%)
Smoke but not at home	11 (19.6%)	3 (5.8%)	14 (13.0%)
Ex-smoker	0 (0.0%)	3 (5.8%)	3 (2.8%)
Never	44 (78.6%)	45 (86.5%)	89 (82.4%)
Number of cigarettes (cigarettes/year)			
Father	2,990.4 (2,681.9, 12–11,520)	3,223.3 (3,112, 0–8,760)	3,101.7 (2,388.6, 12–11,520)
Mother	1,825 (-,1,825)	1,525 (-,1,525)	1,675 (212.1, 1,525–1,825)
Job			
Father			
Managers	3 (5.4%)	3 (5.8%)	6 (5.6%)
Professionals	8 (14.3%)	7 (13.5%)	15 (13.9%)
Technicians and associate professionals	3 (5.4%)	3 (5.8%)	6 (5.6%)
Clerical and support workers	2 (3.6%)	2 (3.8%)	4 (3.7%)
Service and sales workers	13 (23.2%)	17 (32.7%)	30 (27.8%)
Craft and related trades workers	8 (14.3%)	7 (13.5%)	15 (13.9%)
Plant and machine operators and assemblers	9 (16.1%)	6 (11.5%)	15 (13.9%)
Elementary occupations	9 (16.1%)	7 (13.5%)	16 (14.8%)
Armed forces occupations	1 (1.8%)	0 (0%)	1 (0.9%)
Mother			
Manager	0 (0.0%)	1 (1.9%)	1 (0.9%)
Professionals	2 (3.6%)	8 (15.4%)	10 (9.3%)
Technician and associate professionals	0 (0.0%)	0 (0.0%)	0 (0.0%)
Clerical support workers	2 (3.6%)	0 (0.0%)	2 (1.9%)
Service and sales workers	5 (8.9%)	7 (13.5%)	12 (11.1%)
Craft and related trades workers	7 (12.5%)	9 (17.3%)	16 (14.8%)
Plant and machine operators and assemblers	0 (0.0%)	1 (1.9%)	1 (0.9%)
Elementary occupations	2 (3.6%)	1 (1.9%)	3 (2.8%)
Armed forces occupations	0 (0.0%)	0 (0.0%)	0 (0.0%)
Unemployed	38 (67.9%)	25 (48.1%)	63 (58.3%)

^a^ mean (SD, range) or number (%).

**Table A3:** Home environmental conditions of the study children.

Variable ^a^	Males	Females	Total
Distance house to school (m)	766.8 (678.2,3–3.000)	993.9 (1.090.3,3–6.000)	876.1 (903.3,3–6.000)
Time length to school (minutes)	10.9 (8.9, 2–60)	9.1 (4.7, 2–20)	10.1 (7.2, 2–60)
House near street (m)	380.5 (10–3,000)	281.5 (2–1,500)	332.8 (2–3,000)
Transportation to school			
Foot	24 (42.9%)	24 (42.9%)	48 (44.4%)
Bus	3 (5.4%)	2 (3.6%)	5 (4.6%)
Motorcycle	23 (41.1%)	21 (37.5%)	44 (40.7%)
Car	1 (1.8%)	4 (7.1%)	5 (4.6%)
Bicycle	5 (8.9%)	1 (1.8%)	6 (5.7%)
Drinking water sources			
Well water	29 (51.8%)	25 (48.1%)	54 (50.0%)
Tap water	4 (7.1%)	3 (5.8%)	7 (6.5%)
Bottled mineral water	23 (41.1%)	24 (46.2%)	47 (43.5%)
Home painted	52 (92.9 %)	47 (90.4 %)	99 (91.7 %)
Peeled paint at home	25 (44.6 %)	26 (50 %)	51 (47.2 %)
Home near factory	17 (30.4 %)	19 (36.5 %)	36 (33.3 %)
Plastic toys use	44 (78.6%)	39 (75.0%)	83 (76.9%)
Canned food/drink consumption	26 (46.4%)	28 (53.8%)	54 (50.0%)
Traditional medicine	27 (48.2%)	23 (44.2%)	50 (46.3%)
consumption			

^a^ mean (SD, range) or number (%).
